# Deubiquitinase USP29 Governs MYBBP1A in the Brains of Parkinson’s Disease Patients

**DOI:** 10.3390/jcm9010052

**Published:** 2019-12-24

**Authors:** Areum Jo, Yunjong Lee, Chi-Hu Park, Joo-Ho Shin

**Affiliations:** 1 Division of Pharmacology, Department of Molecular Cell Biology, Sungkyunkwan University School of Medicine, Suwon 16419, Korea; alm7760@gmail.com (A.J.); ylee69@skku.edu (Y.L.); 2 Single Cell Network Research Center, Sungkyunkwan University School of Medicine, Suwon 16419, Korea; 3 Samsung Biomedical Research Institute, Samsung Medical Center, Seoul 06351, Korea; 4 Natural Bioactive & Anticancer Research Institute, YEPBio Co., Ltd., Suwon 16229, Korea; chihupark@naver.com

**Keywords:** Parkinson’s disease, parkin, FBP1, AIMP2, USP29, MYBBP1A

## Abstract

The inactivation of parkin by mutation or post-translational modification contributes to dopaminergic neuronal death in Parkinson’s disease (PD). The substrates of parkin, FBP1 and AIMP2, are accumulated in the postmortem brains of PD patients, and it was recently suggested that these parkin substrates transcriptionally activate deubiquitinase *USP29*. Herein, we newly identified 160 kDa myb-binding protein (MYBBP1A) as a novel substrate of USP29. Knockdown of parkin increased the level of AIMP2, leading to ultimately USP29 and MYBBP1A accumulation in SH-SY5Y cells. Notably, MYBBP1A was downregulated in the ventral midbrain (VM) of *Aimp2* knockdown mice, whereas the upregulation of MYBBP1A was observed in the VM of inducible *AIMP2* transgenic mice, as well as in the substantia nigra of sporadic PD patients. These results suggest that AIMP2 upregulates USP29 and MYBBP1A in the absence of parkin activity, contributing to PD pathogenesis.

## 1. Introduction

*Parkin* (*PARK2*) E3 ubiquitin ligase has been identified as the causative gene for familial early-onset Parkinsonism [1]. According to the Parkinson Disease Mutation Database (PDmutDB, http://www.molgen.vib-ua.be/PDmutDB), 127 mutations of parkin have been assigned as pathogenic mutations, accounting for autosomal recessive Parkinsonism. The extensive studies have revealed that the role of parkin is not limited to the proteasomal degradation of specific substrates, but it is also involved in nondegradative ubiquitination in a variety of processes, such as receptor trafficking and mitochondrial quality control [2,3,4].

To date, a number of putative parkin substrates have been identified [5]. Among them, aminoacyl-tRNA synthetase-interacting multifunctional protein type 2 (AIMP2/p38/JTV-1) and fuse-binding protein 1 (FBP1) are considered to be pathogenic substrates of parkin due to their accumulation in *parkin* knockout (KO) mice and in postmortem brains from autosomal recessive PD patients or sporadic cases [6,7]. Our previous study showed that transgenic mice overexpressing *AIMP2* in the brain (Tg-AIMP2) show age-dependent, selective loss of dopaminergic neurons (DA) along with motor deficits [8]. Notably, the neuronal loss caused by AIMP2 overexpression is found in the substantia nigra (SN) but not in cortical neurons of Tg-AIMP2, indicating that AIMP2 might play a toxic role in a region-specific manner [8]. AIMP2 interacts with poly(ADP-ribose)-polymerase-1 (PARP1) and hyper-activates PARP1 to produce poly(ADP-ribose) polymers, ultimately triggering caspase-independent cell death [8,9]. However, the administration of a PARP inhibitor partially prevents AIMP2-mediated dopaminergic neuronal death, suggesting that AIMP2 accumulation might be involved in another mechanism underlying PD neurodegeneration. A recent study revealed that AIMP2 translocated to the nucleus, associated with FBP1, and co-activated the transcription of *ubiquitin-specific peptidase 29* (*USP29*) in oxidative conditions [10].

The nucleolar protein Myb-binding protein 1α (MYBBP1A) is known for enhancing p53 tetramerization by directly interacting with p53 [11]. Recently, MYBBP1A was identified as a binding partner of PARIS (parkin-interacting substrate, ZNF746), which suppresses rRNA transcription and increased p53, a molecular marker of nucleolar stress, in the SN of conditional parkin KO mice and in sporadic PD patients [12].

Since these studies suggest that there might be a pathological relationship between USP29 and MYBBP1A in PD pathogenesis, we investigated whether USP29 regulates MYBBP1A in the absence of parkin. In this study, we identified the 160 kDa myb-binding protein (MYBBP1A) as a novel substrate of USP29. The upregulation of USP29 and MYBBP1A was recorded in the ventral midbrain (VM) of Tg-AIMP2 mice, as well as sporadic PD patients. These results might suggest that the loss of parkin transcriptionally upregulates USP29 via AIMP2 accumulation, leading to an increase of MYBBP1A in PD post-mortem brains.

## 2. Materials and Methods

### 2.1. Cell Culture and Transfection

Human neuroblastoma SH-SY5Y cells (KCLB, https://cellbank.snu.ac.kr) were grown in DMEM containing 10% FBS and antibiotics in a humidified 5% CO_2_/95% air atmosphere at 37 °C. For transient transfection, cells were transfected with indicated amounts of target vector using X-tremeGENE™ HP (Roche, Basel, Switzerland) according to the manufacturer’s instructions. For overexpression experiments, SH-SY5Y cells were transfected with 2 µg of each DNA (Flag-MYBBP1A, HA-USP29, and HA-Ub) to 5 × 10^5^ cells in a 60 mm dish. For the knockdown (KD), 1 µg of shRNA-Parkin, 10 pmol of siRNA-USP29, and siRNA-control were transfected to 2 × 10^5^ cells in a 12 well plate.

### 2.2. Ubiquitination Assay

For the ubiquitination assay, SH-SY5Y cells were transiently transfected with 2 µg of Flag-MYBBP1A generously provided by Dr. Attila Németh (University of Regensburg, Regensburg, Germany) [13], HA-USP29 given by Dr. David Levens (National Cancer Institute, Bethesda, MD, USA) [10], and pMT123-HA-ubiquitin plasmids for 48 h.

### 2.3. Sample Preparation

Mice were euthanized by cervical dislocation. Mouse brain subregions (cerebral cortex (CTX), VM, and striatum (STR)) were harvested as described previously [14]. For immunoprecipitation with mouse brain (SN, STR, CTX, and cerebellum (CB)) and human PD brain (SN and CTX), tissues were homogenized in RIPA buffer (Pierce, Waltham, USA), and Complete Protease Inhibitor Mixture (Roche, Basel, Switzerland). After homogenization (Tissue Grinder, DWK Life Sciences, Mainz, Germany), samples were rotated at 4 °C for 30 min until complete lysis was achieved, after which the homogenate was centrifuged at 14,400 rpm for 20 min, and the supernatant was collected. Protein levels were quantified using the BCA kit (Pierce, Waltham, MA, USA) with BSA standards.

### 2.4. Immunoprecipitation and Immunoblot Anlysis

For immunoprecipitation experiments, SH-SY5Y cells were transfected with the indicated plasmids. Two days after transfection, cells were washed with ice-cold phosphate-buffered saline (PBS) and harvested by centrifugation at 3000× *g* for 10 min at 4 °C. After centrifugation, the pellet was resuspended in RIPA buffer (25 mM Tris-HCl pH 7.6, 150 mM NaCl, 1% NP-40, 1% sodium deoxycholate, 0.1% SDS, Pierce, Waltham, MA, USA) containing protease inhibitors. After centrifugation at 13,000 × *g* for 10 min at 4 °C, the supernatant was cleared by incubation with 50 µl of protein A/G-agarose beads (Santa Cruz, Dallas, TX, USA) for at least 1 h at 4 °C. PBS was added into pre-cleared lysate at a 1:1 ratio to dilute SDS. The pre-cleared supernatant was incubated with protein A/G-agarose beads and the indicated antibodies overnight at 4 °C. The beads were then washed three times with ice-cold RIPA buffer and resuspended with 2× SDS sample buffer (Biorad, Hercules, CA, USA). The precipitates were subject to immunoblot analysis.

Total lysate, brain lysate, and immunopurified proteins were electrophoresed in SDS-polyacrylamide, transferred to nitrocellulose membrane (Biorad), and probed with specific antibodies. The following antibodies were used: anti-Flag (#F1804, Sigma, St. Louis, MO, USA), anti-HA (#H6908, Sigma), anti-β-actin (#ab49900, Abcam, Cambridge, United Kingdom), anti-MYBBP1A (#ab99361, Abcam), anti-USP29 (#ab108056, Abcam), anti-HSP90 (#ab13492, Abcam), anti-VDAC1 (#4866, Cell signaling, Danvers, MA, USA), anti-SP1 (#ab77441, Abcam), anti-Histone H3 (#9715, Cell signaling), anti-Ubiquitin (#sc-8017, Santa Cruz), anti-Parkin (#4211, Cell signaling), anti-FBP1 (#sc-393928, Santa Cruz), and anti-AIMP2 (#10424-1-AP, Proteintech, Rosemont, IL, USA). Each primary antibody was diluted 1:3000.

### 2.5. Cycloheximide Chase Assay

SH-SY5Y cells were seeded in a 12 well plate (2 × 10^5^ cells per well) and transfected with the indicated DNAs (Flag-MYBBP1A, HA-USP29, siRNA-USP29). After 12 h of DNA transfection, cycloheximide (dissolved in DMSO, 100 μg/mL) was added to cells to block protein synthesis. Cells were harvested after treatment for a given time (0, 2, 4, 6, 8, or 12 h) and lysed in RIPA buffer. The results of the chase assay were analyzed by immunoblot.

### 2.6. Quantitative RT-PCR

Total RNA was extracted from SH-SY5Y cells, mouse brain (SN), and PD brain (STR) using the easy-spin total RNA extraction kit (iNtRON, Seongnam-si, Korea). cDNA was synthesized from total RNA (3 μg) using a First-strand cDNA synthesis kit (Invitrogen, Carlsbad, CA, USA). Real-time qRT-PCR was performed using a RotorgeneQ (Qiagen, Hilden, Germany) and Rotorgene SYBR green PCR kit (Qiagen, Hilden, Germany). The primer sequences used were human *USP29*, 5′-GGCTCTCCAGGGTCCTTATC-3′ (forward), 5′-TCAAGGGAAGAGGTGGTTTG-3′ (reverse); mouse *Usp29* 5′-GCAGGAAGATGACCCACATT-3′ (forward), 5′-CCTGAATGGAGGGATCTGAA-3′ (reverse); mouse *Aimp2*, 5′-AACGCTTGTATGAGTTGAAGGC-3′ (forward), and 5′-TCTGGGGTGTGAATCATCTTTG-3′ (reverse).

### 2.7. RNAi

MISSION short hairpin RNA (shRNA) plasmid targeting parkin (#TRCN0000000285) was purchased from Sigma (St. Louis, MO, USA). siRNA-USP29 (siUSP29) (#sc-76833) and control siRNA (#sc-37007) were purchased from Santa Cruz. A total of 2 × 10^5^ cells/well in a 12 well plate were transfected with 10 pmol each siRNA using Lipofectamine RNAiMAX Reagent (Thermo Scientific, Waltham, MA, USA) was used as per the manufacturer’s instructions. RNA interference efficiency was confirmed by western blotting.

### 2.8. Subcellular Fractionation

A subcellular protein fractionation kit (#78840, Thermo Scientific, Waltham, MA, USA) was used for subcellular fractionation of SH-SY5Y cells into the cytoplasm, membrane, soluble nuclear, and chromatin-bound fractions following the manufacturer’s instructions.

### 2.9. Brain Specimens of Mouse Line and PD Patients

Conditional transgenic *AIMP2* mouse model (Tg-AIMP2) was generated by cross-breeding *TetP-AIMP2* mice with *CamkIIα-tTA* driver mice [8]. Three month old Tg-AIMP2 mice were used for biochemical experiments. All animal experiments were approved by the Sungkyunkwan University Ethical Committee in accordance with international guidelines. The brains of *Aimp2* knockdown (KD) mice were provided by Dr. Kim S. (Seoul National University, Seoul, Korea) [15]. Mutations in the mouse genomic DNA were generated by the gene trap method [16]. Information for SN and STR tissues was provided in a previous study [17]. PD cortex specimens were supplied from the Banner Sun Health Research Institute’s (BSHRI, Sun City, AZ, USA) Brain and Body Donation Program (BBDP).

### 2.10. Quantification and Statistical Analysis

For immunoblot analysis, densitometric analysis of the bands was performed using ImageJ (NIH, Bethesda, MD, USA, http://rsb.info.nih.gov/ij/). The intensities of protein bands were normalized using β-actin as a loading control. Statistical analyses were performed using GraphPad Prism version 7 (GraphPad Software). Data were determined to be statistically significant when *p* < 0.05 by applying the unpaired two-tailed Student’s *t*-test (for comparison between two groups) or one-way ANOVA test with Tukey’s post-hoc test (for comparison among three groups or more). Average values obtained from experiments are indicated at the top of the graphs. In figures, asterisks denote statistical significance as calculated by Student’s t-test or ANOVA test (* *p* < 0.05, ** *p* < 0.01, and *** *p* < 0.001) as compared to controls, unless otherwise specified by lines connecting the compared pieces of data.

## 3. Results

### 3.1. USP29 Binds to MYBBP1A

**Figure 1:**
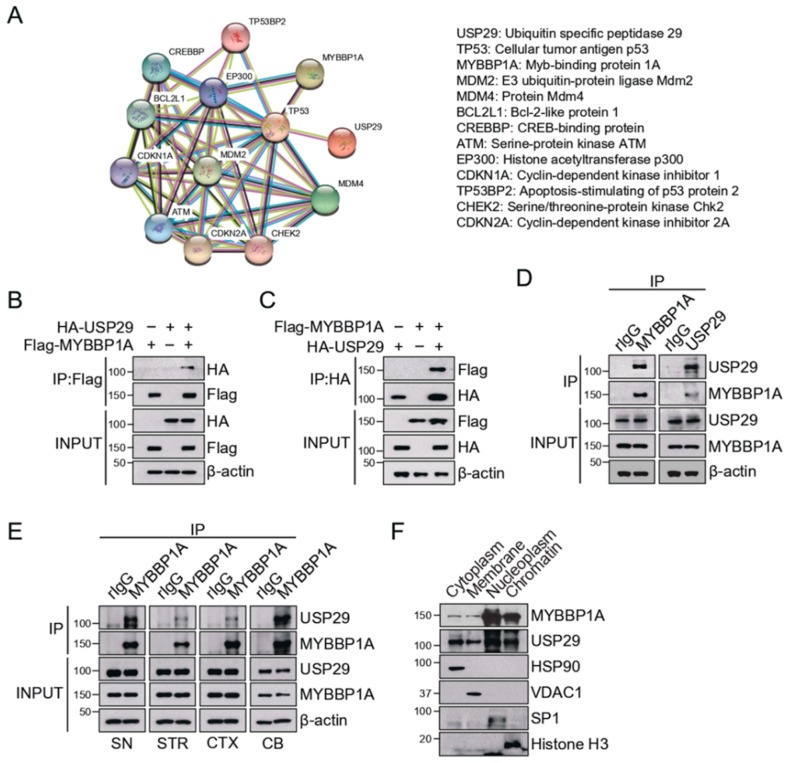
USP29 binds to MYBBP1A. (**A**) USP29 protein functional association network by STRING. An edge was drawn with differently colored lines representing the existence of different types of evidence. (**B**,**C**) Co-immunoprecipitated Flag-MYBBP1A interacts with HA-USP29. (**D**) Co-immunoprecipitated MYBBP1A and USP29 in SH-SY5Y cells. Rabbit IgG (rlgG) was used as a negative control. (**E**) Immunoprecipitated MYBBP1A interacts with USP29 in substantia nigra (SN), striatum (STR), cerebral cortex (CTX), and cerebellum (CB) of C57BL mouse. rlgG was used as a negative control. (**F**) Subcellular localization of MYBBP1A and USP29 in SH-SY5Y cells.

To expand our knowledge about USP29 and to identify novel substrate of USP29, we utilized a database and literature search, which revealed that USP29 has been experimentally proven to interact with p53 (STRING, http://string-db.org) (Figure 1A). Since USP29 stabilizes p53 [10] and MYBBP1A is required for p53 acetylation and tetramerization in the process of nucleolar disruption [11,18], we hypothesized that USP29 might be involved in proteasomal regulation of MYBBP1A.

To confirm whether USP29 binds to MYBBP1A, Flag-tagged MYBBP1A and HA-tagged USP29 were co-transfected into SH-SY5Y cells and Flag-tagged MYBBP1A was immunoprecipitated, showing that Flag-MYBBP1A pulled down HA-USP29 (Figure 1B). In addition, another immunoprecipitation assay showed their strong interaction in the other direction (Figure 1C). For validating the endogenous interaction of the two proteins, endogenous MYBBP1A was immunoprecipitated, indicating that MYBBP1A successfully pulled down endogenous USP29 in SH-SY5Y (Figure 1D). Furthermore, their interaction was confirmed in the SN, STR, CTX, and CB of the mouse brain by immunoprecipitation assay. (Figure 1E). We observed that there was no brain-region-specific interaction between USP29 and MYBBP1A, indicating that MYBBP1A might be an authentic physiological substrate of USP29 (Figure 1E). In addition, the subcellular fractionation assay showed that USP29 and MYBBP1A were mainly localized in the nucleus and chromatin-bound fractions (Figure 1F).

### 3.2. USP29 Regulates MYBBP1A Levels via Deubiquitination

**Figure 2:**
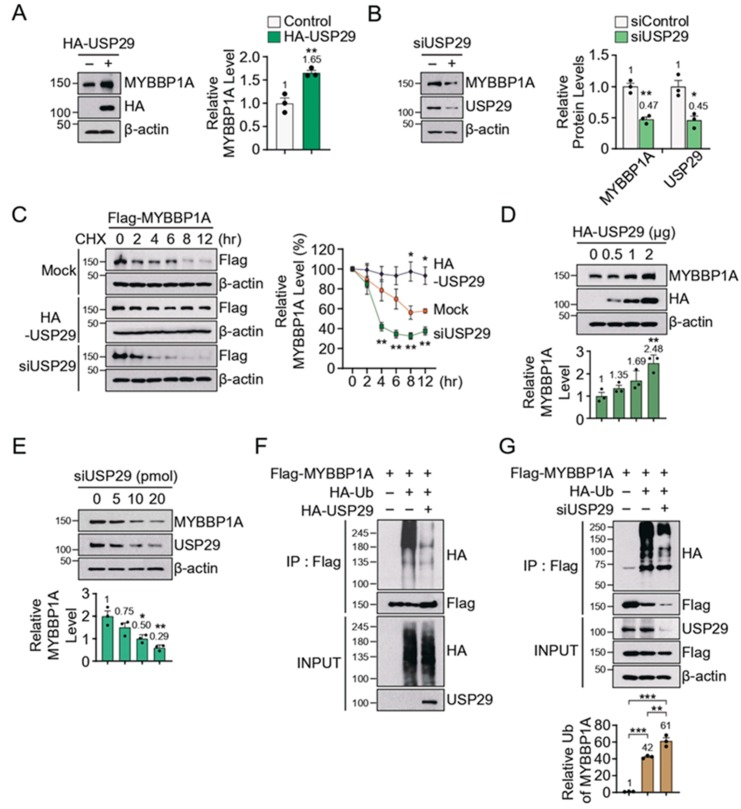
USP29 regulates MYBBP1A via deubiquitination. (**A**) Immunoblot analysis of MYBBP1A in USP29 overexpressed cells normalized to β-actin; *n* = 3 independent experiments. Quantitation of the immunoblots in the right panel. (**B**) Immunoblot analysis of MYBBP1A in *USP29* knockdown cells normalized to β-actin; *n* = 3 independent experiments. Quantitation of the immunoblots in the right panel. (**C**) Relative Flag-MYBBP1A protein level of SH-SY5Y cells transfected with HA-USP29 or siRNA-USP29 treated with cycloheximide (CHX, 100 μg/mL) in a time-dependent manner; *n* = 3 independent experiments. Quantitation of the immunoblots in the right panel. (**D**) Relative MYBBP1A protein level of SH-SY5Y cells transfected HA-USP29 in a dose-dependent manner; *n* = 3 independent experiments. Quantitation of the immunoblots in the bottom panel. (**E**) Relative MYBBP1A protein level of SH-SY5Y cells transfected siRNA-USP29 (siUSP29) in a dose-dependent manner; *n* = 3 independent experiments. Quantitation of the immunoblots in the bottom panel. (**F**) Representative immunoblots of ubiquitin and Flag from anti-Flag IP samples of MYBBP1A-expressing SH-SY5Y with HA-USP29. (**G**) Representative immunoblots of ubiquitin and Flag from anti-Flag IP samples of MYBBP1A expressed SH-SY5Y with siRNA-USP29. Quantitation of the ubiquitin of MYBBP1A in the bottom panel. Data are expressed as mean ± SEM. Statistical significance was evaluated by applying an unpaired two-tailed Student’s t-test (A–C) or using one-way ANOVA with Tukey’s post-hoc test (D,E,G). Differences are considered significant when *p* < 0.05. * *p* < 0.05, ** *p* < 0.01, and *** *p* < 0.001.

In order to investigate whether USP29 regulates the protein level of MYBBP1A, we transfected HA-tagged USP29 and monitored the endogenous level of MYBBP1A in SH-SY5Y cells. Overexpression of USP29 led to an approximately 1.7-fold increase of MYBBP1A (Figure 2A), while the knockdown of USP29 resulted in a significant decrease of MYBBP1A (Figure 2B). Next, we monitored the steady-state level of MYBBP1A in SH-SY5Y cells transfected with either HA-tagged USP29 or siRNA-USP29 (siUSP29) by cycloheximide (CHX) chase assay (Figure 2C). The decrease in the steady-state levels of MYBBP1A was accelerated in the absence of USP29, while degradation of MYBBP1A was inhibited by the overexpression of USP29 (Figure 2C). Moreover, the upregulation of endogenous MYBBP1A was dependent upon USP29 overexpression, whereas endogenous MYBBP1A was gradually reduced by USP29 knockdown (Figure 2D,E). These results indicate that USP29 tightly regulates the steady-state level of MYBBP1A.

In vitro ubiquitin assay demonstrated that immunoprecipitated Flag-tagged MYBBP1A showed a smear pattern of ubiquitin (Ub) immunoreactivity, which is the indicator of polyubiquitination (Figure 2F). Overexpression of USP29 significantly reduced the ubiquitin signal of MYBBP1A, leading to the accumulation of Flag-MYBBP1A (Figure 2F). In contrast, knockdown of *USP29* resulted in a decrease of MYBBP1A. Reduced HA immunoreactivity of MYBBP1A was attributed to proteasomal degradation of ubiquitinated MYBBP1A. Indeed, the relative amount of HA immunoactivity normalized by immunoprecipitated Flag signal was increased in the lack of USP29 (Figure 2G). Taken together, these data suggest that USP29 hydrolyzes the polyubiquitin chain on MYBBP1A.

### 3.3. Increased Levels of USP29 and MYBBP1A in PD Models

**Figure 3:**
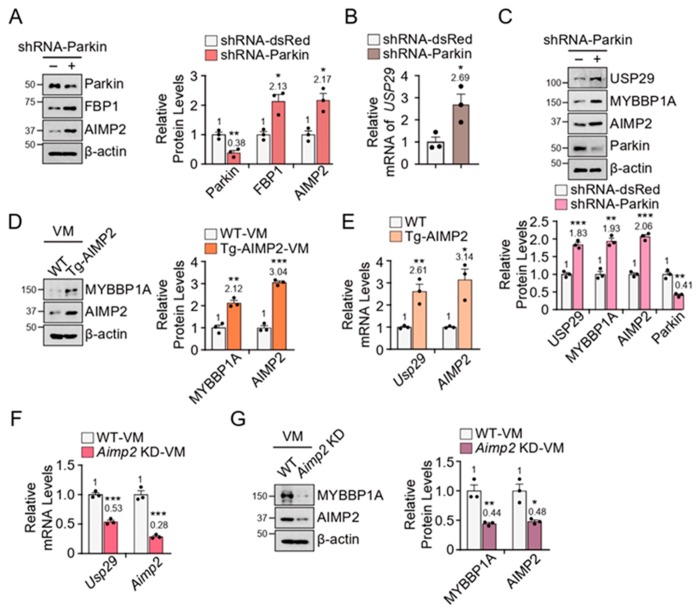
Increased USP29 and MYBBP1A in Parkinson’s disease (PD) models. (**A**) Immunoblot analysis of parkin, FBP1, and AIMP2 in *parkin* knockdown (KD) cells normalized to β-actin; *n* = 3 independent experiments. Quantitation of the immunoblots in the right panel. (**B**) Relative mRNA level of *USP29* normalized to *β-actin* by real-time qRT-PCR in *parkin* KD cells; *n* = 3 mice per group. (**C**) Immunoblot analysis of USP29, MYBBP1A, AIMP2, and parkin in *parkin* KD cells normalized to β-actin, *n* = 3 independent experiments. Quantitation of the immunoblots in the bottom panel. (**D**) Expression of MYBBP1A and AIMP2 in wild-type (WT) and AIMP2 transgenic (Tg-AIMP2) mouse VM; *n* = 3 independent experiments. Quantitation of the immunoblots in the right panel. (**E**) Relative mRNA levels of *Usp29* and *AIMP2* genes normalized to *β-actin* by real-time qRT-PCR in the VM of Tg-AIMP2 mice; *n* = 3 mice per group. (**F**) Relative mRNA levels of *Usp29* and *Aimp2* genes normalized to *β-actin* by real-time qRT-PCR in the VM of *Aimp2* KD mice; *n* = 3 mice per group. (**G**) Expression of MYBBP1A and AIMP2 in *Aimp2* WT and KD mice VM; *n* = 3 independent experiments. Quantitation of the immunoblots in the right panel. Data = mean ± SEM. Statistical significance was determined by applying an unpaired two-tailed Student’s t-test. Differences were considered significant when *p* < 0.05. * *p* < 0.05, ** *p* < 0.01, and *** *p* < 0.001.

Since the loss of parkin leads to the accumulation of authentic substrates AIMP2 and FBP1 in PD, we next evaluated the levels of USP29 and MYBBP1A in the absence of parkin. shRNA-mediated *parkin* knockdown in SH-SY5Y cells successfully resulted in the upregulation of AIMP2 and FBP1 proteins (Figure 3A), as well as the robust induction of the *USP29* messenger (Figure 3B). Accompanying the accumulation of AIMP2 in the absence of parkin was the upregulation of USP29 and MYBBP1A (Figure 3C).

To investigate whether the accumulation of AIMP2 increased *USP29* transcription *in vivo*, we utilized a conditional transgenic *AIMP2* mouse model (Tg-AIMP2) generated by cross-breeding *TetP-AIMP2* mice with *CamkIIα-tTA* driver mice [8].

*AIMP2* was induced at 3 months of age by withdrawal of doxycycline diet. Immunoblot analysis showed the increase of AIMP2 and MYBBP1A in the VM of Tg-AIMP2 (Figure 3D). Real-time qRT-PCR analysis confirmed the overexpression of *AIMP2* in the VM of Tg-AIMP2 mice (Figure 3E). Accordingly, the mRNA level of *Usp29* was upregulated in the VM of Tg-AIMP2 mice (Figure 3E).

In order to confirm the correlation between MYBBP1A and AIMP2, USP29 and MYBBP1A levels were monitored in the VM of *Aimp2* knockdown (*Aimp2* KD) mice. The downregulated mRNA levels of *Usp29* and *Aimp2* were observed in the VM of *Aimp2* KD mice (Figure 3F). Consistent with these results, MYBBP1A was downregulated in the VM of *Aimp2* KD mice (Figure 3G).

### 3.4. MYBBP1A Upregulation in PD Patients

**Figure 4:**
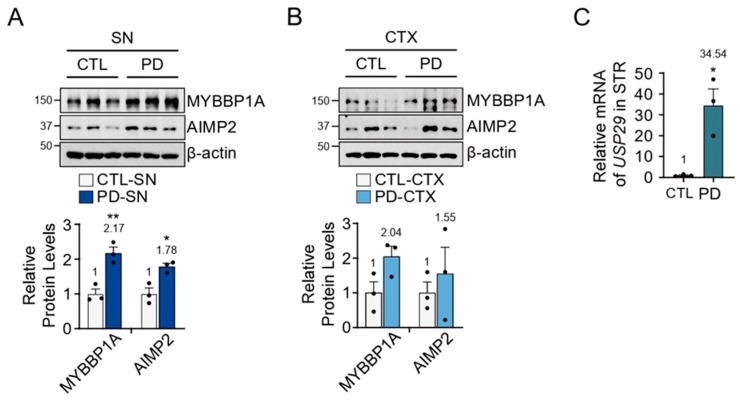
MYBBP1A was increased in PD brains. (**A**) MYBBP1A and AIMP2 levels in the SN of sporadic PD patient brains as compared to age-matched controls; *n* = 3 per group. Quantification of proteins was normalized to β-actin, bottom panel. (**B**) Immunoblot analysis of MYBBP1A and AIMP2 in the CTX from sporadic PD patients compared to age-matched controls; *n* = 3 per group. Quantification of proteins was normalized to β-actin, bottom panel. (**C**) Relative mRNA levels of *USP29* gene normalized to *β-actin* by real-time qRT-PCR in the STR from sporadic PD patients compared to age-matched controls; *n* = 3 per group. Data = mean ± SEM. Statistical significance was determined by applying an unpaired two-tailed Student’s t-test. Differences were considered significant when *p* < 0.05. * *p* < 0.05 and ** *p* < 0.01.

To evaluate the relationship of the MYBBP1A–USP29 pathway with PD pathogenesis, we investigated the protein levels of MYBBP1A and AIMP2 in postmortem PD brains. We observed a significant upregulation of MYBBP1A and AIMP2 accumulation in the SN of PD patients (Figure 4A). To understand whether this event occurred in a brain-region-specific manner, we monitored the levels of MYBBP1A and AIMP2 protein in the CTX of PD patients, revealing there was an increasing trend of MYBBP1A in the CTX of PD patients (Figure 4B). Due to the unavailability of cDNA from SN, we were only able to investigate the messenger level of *USP29* in the STR of PD patients, showing significant upregulation of *USP29* mRNA in PD brain (Figure 4C). These data suggest that USP29 and MYBBP1A are upregulated under pathogenic conditions.

## 4. Discussion

Previously, we reported that PARIS interacts with MYBBP1A and suppresses rRNA transcription in the SN of conditional *parkin* KO mice and PD patients [12]. In addition, a recent study revealed that AIMP2–FBP1 complex transcriptionally activates *USP29* in oxidative conditions [10]. Since PARIS, AIMP2, and FBP1 have been identified as authentic substrates of parkin, we hypothesized that there might be a pathological relationship between USP29 and MYBBP1A in the loss of parkin.

In this study, we identified MYBBP1A as a new substrate of USP29. Reduction of USP29 results in MYBBP1A destabilization, whereas its overexpression increases MYBBP1A level via deubiquitination. In addition, we showed that the AIMP2 and FBP1 proteins were accumulated in the loss of parkin, leading to the upregulation of USP29 and MYBBP1A in SH-SY5Y cells. Accordingly, the level of MYBBP1A was significantly increased in the SN of PD brains, suggesting that the increase of USP29 might be involved in PD pathogenesis. Despite little being known about USP29, USP29 has been shown to deubiquitinate and stabilize p53, causing apoptosis in oxidative stress [10]. Recently, it was demonstrated that USP29 controls the level of Claspin, which is a key player in the ATR–Chk1 pathway of the DNA damage checkpoint and is also required for normal DNA replication [19,20,21].

As mentioned, our group previously reported that PARIS interacts with MYBBP1A [12]. PARIS and MYBBP1A independently repress rRNA transcription and trigger nucleolar stress, leading to the activation of p53 by acetylation [21]. PARIS and MYBBP1A have also been found to act as transcriptional suppressors of *peroxisome-proliferator-activated receptor gamma coactivator 1-alpha* (*PGC-1α*) [17,22]. These results, taken together, indicate that the toxicity of PARIS in PD models might be attributable not only to transcriptional suppression of *PGC-1α*, but also to the inhibition of *PGC-1α* by MYBBP1A upregulation and p53 activation.

Based on this evidence, the loss of parkin might lead to the occurrence of nucleolar stress and mitochondrial dysfunction, ultimately triggering dopaminergic neuronal death in PD pathogenesis. It seems that the accumulation of AIMP2 and FBP1 proteins in the absence of parkin is required to increase MYBBP1A level by USP29 upregulation. Interestingly, Harper’s group demonstrated the interaction landscape of human deubiquitinating enzyme and identified MYBBP1A as a USP29 interactor by mass spectrometry [23]. Furthermore, a significant increase of MYBBP1A was observed in the SN of conditional *parkin* KO mice at 9 months post-injection when DA toxicity was observable [12], suggesting that increased MYBBP1A by parkin deficits might contribute to DA neuronal death through *USP29* induction via the AIMP2–FBP1 complex.

## 5. Conclusions

Under PD pathogenic conditions, parkin inactivation by cellular stresses leads to the accumulation of AIMP2, resulting in transcriptional activation of *USP29*. Next, USP29 stabilizes MYBBP1A via deubiquitination in SH-SY5Y cells. Upregulation of USP29 and MYBBP1A was found in *parkin* knockdown SH-SY5Y cells and *AIMP2* transgenic mouse SN, suggesting that the increase of USP29 and MYBBP1A might be associated with PD pathogenesis.

## Abbreviations

PD	Parkinson’s disease
FBP1	Far-upstream element (FUSE) binding protein
AIMP2	Aminoacyl-tRNA synthetase complex-interacting multifunctional protein 2
USP29	Ubiquitin Specific Peptidase 29
MYBBP1A	160-kDa Myb-binding protein 1α
